# The Ottawa Statement on the Ethical Design and Conduct of Cluster Randomized Trials

**DOI:** 10.1371/journal.pmed.1001346

**Published:** 2012-11-20

**Authors:** Charles Weijer, Jeremy M. Grimshaw, Martin P. Eccles, Andrew D. McRae, Angela White, Jamie C. Brehaut, Monica Taljaard, Ottawa Ethics of Cluster Randomized Trials Consensus Group, Fernando Althabe, Ariella Binik, Judith Belle Brown, Robert Boruch, Jamie C. Brehaut, Shazia Chaudhry, Allan Donner, Geneviéve Dubois-Flynn, Martin P. Eccles, Sarah Edwards, Diana Elbourne, Sandra Eldridge, David Forster, Antonio Gallo, Jeremy M. Grimshaw, Catarina Kiefe, Jonathan Kimmelman, Melody Lin, Elizabeth Loder, Kathleen Lohr, Andrew D. McRae, Eileen S. Naughton, Rex J. Polson, Raphael Saginur, Abha Saxena, Julie Spence, Monica Taljaard, Charles Weijer, Angela White, Gerald White, Merrick Zwarenstein

**Affiliations:** Institute for Clinical Effectiveness and Health Policy, Buenos Aires, Argentina; Rotman Institute of Philosophy, London, Canada; Western University, London, Canada; University of Pennsylvania, Philadelphia, Pennsylvania, US; Ottawa Hospital Research Institute, Ottawa, Canada; University of Ottawa, Ottawa, Canada; Western University, London, Canada; Canadian Institutes for Health Research Ethics Office, Ottawa, Canada; Newcastle University, Newcastle upon Tyne, UK; University College London, London, UK; London School of Hygiene and Tropical Medicine, London, UK; Queen Mary University of London, London, UK; Western Institutional Review Board, Olympia, Washington, US; Rotman Institute of Philosophy, London, Canada; Ottawa Hospital Research Institute, Ottawa, Canada; University of Massachusetts Medical School, Worcester, Massachusetts, US; McGill University, Montreal, Canada; Office for Human Research Protections, Rockville, Maryland, US; Loder (Harvard Medical School, Boston, Massachusetts, US; RTI International, Research Triangle Park, North Carolina, US; University of Calgary, Calgary, Canada; Rhode Island House of Representatives, Providence, Rhode Island, US; Solihull Hospital, Solihull, UK; Ottawa Hospital, Ottawa, Canada; World Health Organization, Geneva, Switzerland; St. Michael’s Hospital, Toronto, Canada; Ottawa Hospital Research Institute, Ottawa, Canada; Rotman Institute of Philosophy, London, Canada; Rotman Institute of Philosophy, London, Canada; Health Council of Canada, Toronto, Canada; (Institute for Clinical Evaluative Studies, Toronto, Canada; 1 Rotman Institute of Philosophy, Department of Philosophy, Western University, London, Ontario, Canada; 2 Department of Medicine, Western University, London, Ontario, Canada; 3 Department of Epidemiology and Biostatistics, Western University, London, Ontario, Canada; 4 Clinical Epidemiology Program, Ottawa Hospital Research Institute, Ottawa, Ontario, Canada; 5 Department of Medicine, Faculty of Medicine, University of Ottawa, Ottawa, Ontario, Canada; 6 Institute of Health and Society, Newcastle University, Newcastle upon Tyne, United Kingdom; 7 Division of Emergency Medicine, University of Calgary, Foothills Medical Centre, Calgary, Alberta, Canada; 8 Department of Epidemiology and Community Medicine, University of Ottawa, Ottawa, Ontario, Canada

## Abstract

The Ottawa Ethics of Cluster Trials Consensus Group sets out 15 recommendations for the ethical design and conduct of cluster randomized trials.

**Summary Points**

- In cluster randomized trials (CRTs), the units of allocation, intervention, and outcome measurement may differ within a single trial. As a result of the unique design of CRTs, the interpretation of existing research ethics guidelines is complicated.
- The Ottawa Statement on the Ethical Design and Conduct of Cluster Randomized Trials aims to provide researchers and research ethics committees (RECs) with detailed guidance on the ethical design, conduct, and review of CRTs.
- A five-year mixed methods research project explored the ethical challenges of CRTs. Empirical studies documented the reporting of ethical issues in published CRTs, interviewed experienced trialists, and surveyed trialists and REC chairs. The ethical issues identified were explored in a series of background papers that provided detailed ethical analyses and policy options, and a panel of experts using a systematic process developed a consensus statement.
- The Ottawa Statement sets out 15 recommendations for the ethical design and conduct of CRTs. The recommendations provide guidance on the justification of a cluster randomized design, the need for REC review, the identification of research participants, obtaining informed consent, the role of gatekeepers in protecting group interests, the assessment of benefits and harms, and the protection of vulnerable participants.

## Introduction

Cluster randomized trials (CRTs), also known as group randomized, place-based, or community intervention trials, are increasingly important for the evaluation of interventions in health research [1]–[7]. In CRTs, groups, or “clusters”, of individuals—rather than the constituent individuals themselves—are randomly allocated to study arms, and outcomes are then measured on the individual cluster members. Examples of clusters include medical practices, hospital wards, schools, and communities. CRTs often evaluate complex or multifaceted interventions targeted at the cluster, professionals, or individual cluster members. (See Text S1 for a glossary of terms.)

CRTs pose distinct ethical challenges for several reasons. First, in CRTs the units of allocation, intervention, and outcome measurement may differ in a single trial. For example, in a CRT of teaching a new hand-washing technique to help avoid transmitting infection on hospital wards, the unit of allocation may be the hospital, the intervention may be delivered to health professionals, and data may be collected about or from patients. This has implications for identifying research participants and for informed consent procedures. Second, in some CRTs interventions are administered at the cluster level and thus have the potential to directly or indirectly affect the interests of many individuals associated with a cluster, including those quite remote from the study itself. For example, in a community randomized trial of a mass media advertising campaign to promote smoking cessation, the intervention may potentially affect all community residents as well as visitors and those traveling through the study communities. Third, in CRTs clusters are commonly randomized before it is possible to identify and recruit individuals for informed consent, thus consent to randomization may not be obtained. Fourth, cluster-level study interventions may be difficult or impossible for individuals to avoid, thereby precluding the meaningful refusal of study participation. Fifth, CRTs often employ social groups or organizations as the units of allocation, but current understanding of the moral status of such groups, and of the gatekeepers who speak on their behalf, is incomplete. Sixth, whereas risks to individuals may be minor, the risks to the cluster as a whole or to subgroups may be significant, because risks to the group may be underestimated, and vulnerable subgroups within clusters may be difficult to identify.

Although there is a small but growing literature on the subject [8]–[13], the ethical challenges raised by CRTs have yet to be systematically explored. As a result, researchers and research ethics committees (RECs) currently lack specific guidelines to help them design, conduct, and review CRTs according to internationally accepted ethical standards. Predictably, the lack of comprehensive guidance has resulted in uncertainty and markedly different interpretations as to permissible ethical practices in CRTs, both within and across countries.

The aim of this consensus statement is to provide guidance on the ethical design and conduct of CRTs in health research. This guidance is primarily intended for researchers and RECs. It will also be relevant to other groups such as research funders, policy makers, journal editors, and potential study participants. It builds upon—but does not replace—national and international ethics guidelines for randomized controlled trials and other human research. The consensus statement should be interpreted in light of the laws and regulations of the host country or countries, as well as other applicable international standards.

## Methods

The development of the consensus statement was underpinned by a five-year research project funded by the Canadian Institutes of Health Research [14]. The project used a mixed methods approach incorporating both empirical work and ethical analysis. The empirical work included interviews with key informants, review of published CRTs [15], a survey of trialists, and a survey of REC chairs. Based on the empirical work, as well as the practical experiences of research team members, the team identified six questions specific to CRTs in need of further analysis: How should research participants be identified? From whom, how, and when must informed consent be obtained? Does clinical equipoise apply? How does one determine if the benefits outweigh the risks? Who are gatekeepers, and what are their responsibilities? How ought vulnerable groups be protected [16]? The research team conducted an ethical analysis of each issue, which led to a series of discussion papers laying out principles, policy options, and rationales for proposed ethics guidelines [17]–[20]. The research team posted these papers on a wiki (http://crtethics.wikispaces.com) and publicized the wiki in the discussion papers and surveys.

To develop the consensus statement from this process, the research team organized a two-and-a-half-day meeting of a multidisciplinary expert panel that took place in Ottawa, Canada, in November 2011. The research team identified the constituencies and perspectives that needed to be represented within the expert panel, including ethicists, cluster trialists, consumer representatives, RECs, policy makers, funding agencies, and journal editors. Potential expert panel members were identified by consultation with colleagues, via searches of the relevant literature and the Internet, and from respondents in the key informant interviews, trialist survey, and REC chair survey. In addition to six of the members of the research team, 26 external individuals were approached, of whom 13 agreed to participate. (See Text S2 for a list of the 19-member expert panel.) External members were invited as individuals rather than as representatives of their home organizations.

The research team made the discussion papers available to the expert panel in advance of the meeting. The first day of the consensus process was an open meeting with a simultaneous webcast, attended by individuals from the same constituencies and sources used to identify the expert panel. Eighty people participated in person, and a further 20 participated by webcast. The research team presented the results of the empirical studies and the ethical analyses of the six questions, and three expert discussants and the audience commented on the presentations. The open meeting served to further familiarize the expert panel with the content of the materials developed by the research team, and allowed them to hear issues raised about the materials by the broader audience. Video of the open portion of the consensus meeting is available via YouTube (http://www.youtube.com/user/mtaljaard55).

Over the next one and a half days the members of the expert panel met in closed session to discuss the identified issues and to develop recommendations. The expert panel was chaired by Professor Martin Eccles, an experienced small group leader with expertise in chairing guideline development groups. Initial discussions established the “rules of engagement” for the expert panel process. The expert panel agreed about how debate should be conducted and how they wanted the chair to run the process. The expert panel agreed to achieve consensus, where possible, through discussion and would document disagreements; they did not wish to use a majority voting system. Draft recommendations based upon the background papers were presented to the expert panel, and members were asked to identify issues in need of clarification and discussion. Full discussion of these issues was facilitated by the chair with the aim of achieving consensus on the underlying principles, but not necessarily specific wording. All expert panel members actively participated in the discussion. Some draft recommendations were substantially revised during the process. There were no substantive disagreements requiring presentation of dissenting views.

A writing group, consisting of seven members of the research team, then reviewed the results of the meeting and produced a first draft of the consensus statement. The writing group circulated the draft to the expert panel in December 2011 and asked for comments on both the principles and specific wording of the recommendations. Responses were received from all participants, and a point-by-point response to all comments (available on request) was produced and the draft consensus statement revised accordingly. In February 2012, the writing group posted the revised consensus statement on the wiki and invited the expert panel, participants of the open meeting, respondents in the key informant interviews, trialist survey, and REC chair survey, and other contacts of the research team to comment. Again, the writing group produced a point-by-point response to all comments (available on request) and revised the consensus statement. In June 2012, the final draft of the consensus statement was sent to the expert panel for approval, which was given by all members with no dissention.

## Results

General ethical principles govern the design and conduct of health research (see Text S3 for general ethical principles). The 15 recommendations in the Ottawa Statement consider the application of these ethical principles to the design and conduct of CRTs (see Table 1). The recommendations are intended to guide researchers in the design and conduct of CRTs, and to ensure robust and appropriate review of CRTs by RECs. The recommendations are organized by the six identified ethical issues, and each recommendation is followed by a brief explanation or interpretation.

**Table 1:** Summary of recommendations.

Ethical Issue	Recommendation Number	Recommendation
Justifying the cluster randomized design	1	Researchers should provide a clear rationale for the use of the cluster randomized design and adopt statistical methods appropriate for this design.
REC review	2	Researchers must submit a CRT involving human research participants for approval by a REC before commencing.
Identifying research participants	3	Researchers should clearly identify the research participants in CRTs. A research participant can be identified as an individual whose interests may be affected as a result of study interventions or data collection procedures, that is, an individual (1) who is the intended recipient of an experimental (or control) intervention; or (2) who is the direct target of an experimental (or control) manipulation of his/her environment; or (3) with whom an investigator interacts for the purpose of collecting data about that individual; or (4) about whom an investigator obtains identifiable private information for the purpose of collecting data about that individual. Unless one or more of these criteria is met, an individual is not a research participant.
Obtaining informed consent	4	Researchers must obtain informed consent from human research participants in a CRT, unless a waiver of consent is granted by a REC under specific circumstances.
	5	When participants' informed consent is required, but recruitment of participants is not possible before randomization of clusters, researchers must seek participants' consent for trial enrollment as soon as possible after cluster randomization—that is, as soon as the potential participant has been identified, but before the participant has undergone any study interventions or data collection procedures.
	6	A REC may approve a waiver or alteration of consent requirements when (1) the research is not feasible without a waiver or alteration of consent, and (2) the study interventions and data collection procedures pose no more than minimal risk.
	7	Researchers must obtain informed consent from professionals or other service providers who are research participants unless conditions for a waiver or alteration of consent are met.
Gatekeepers	8	Gatekeepers should not provide proxy consent on behalf of individuals in their cluster.
	9	When a CRT may substantially affect cluster or organizational interests, and a gatekeeper possesses the legitimate authority to make decisions on the cluster or organization's behalf, the researcher should obtain the gatekeeper's permission to enroll the cluster or organization in the trial. Such permission does not replace the need for the informed consent of research participants.
	10	When CRT interventions may substantially affect cluster interests, researchers should seek to protect cluster interests through cluster consultation to inform study design, conduct, and reporting. Where relevant, gatekeepers can often facilitate such a consultation.
Assessing benefits and harms	11	The researcher must ensure that the study intervention is adequately justified. The benefits and harms of the study intervention must be consistent with competent practice in the field of study relevant to the CRT.
	12	Researchers must adequately justify the choice of the control condition. When the control arm is usual practice or no treatment, individuals in the control arm must not be deprived of effective care or programs to which they would have access, were there no trial.
	13	Researchers must ensure that data collection procedures are adequately justified. The risks of data collection procedures must (1) be minimized consistent with sound design and (2) stand in reasonable relation to the knowledge to be gained.
Protecting vulnerable participants	14	Clusters may contain vulnerable participants. In these circumstances, researchers and RECs must consider whether additional protections are needed.
	15	When individual informed consent is required and there are individuals who may be less able to choose participation freely because of their position in a cluster or organizational hierarchy, RECs should pay special attention to recruitment, privacy, and consent procedures for those participants.

### Justifying the Cluster Randomized Design

**Recommendation 1: Researchers should provide a clear rationale for the use of the cluster randomized design and adopt statistical methods appropriate for this design.**

Compared with an individually randomized trial with the same number of individuals, CRTs are inefficient and have less statistical power. This is a result of the tendency for responses of individuals within a cluster to be more similar than the responses of individuals in differing clusters. Furthermore, CRTs are more likely than individually randomized trials to have imbalances across study arms at baseline because they tend to have a smaller number of randomized units (e.g., a median of 21 clusters in total in a review of a random sample of 300 published CRTs [15]). CRTs are also more susceptible to various forms of bias, including selection bias, especially when individual participants need to be identified or enrolled *after* cluster allocation [21]. Given its inherent statistical inefficiency and methodological complexities, the use of cluster as opposed to individual randomization should be clearly justified.

Reasons for adopting the CRT design are diverse, and range from sheer necessity (because the intervention can only be administered at the cluster level) to other scientific, practical, or logistical reasons [1]. Common reasons for cluster randomization include the following: to avoid experimental contamination due to intervention and control participants in the same cluster interacting with each other; to enhance participant compliance or cooperation of investigators; to capture the indirect effects of an intervention against infectious diseases (i.e., the effects of herd immunity); for administrative convenience; or to reduce the costs of administering the intervention across a large geographic area. An inappropriate reason to adopt a CRT is the mistaken belief that the need to seek informed consent can be avoided by using cluster randomization.

Once a clear rationale for the cluster randomized design has been established, investigators should adopt statistical methods appropriate for this design. Because multiple observations from the same cluster are usually positively correlated, standard statistical methods for sample size calculation and data analysis are invalid. Several articles provide appropriate methods for sample size calculation and analysis for CRTs [22]–[27].

### REC Review

**Recommendation 2: Researchers must submit a CRT involving human research participants for approval by a REC before commencing.**

There is broad agreement in national and international research ethics guidelines that all human research should be submitted to and approved by a REC. Whereas the integrity of researchers is an important protection for research participants, researchers may have vested interests. RECs are better placed to ensure that the autonomy and welfare interests of research participants are protected, and that national and international ethics standards are upheld.

Research may usefully be defined as a systematic investigation that is designed to produce generalizable knowledge. Quality improvement initiatives that seek solely to improve local service delivery are (generally) not regarded as research and may not require REC review. However, CRTs, including those evaluating quality improvement and knowledge translation interventions, are clearly designed to produce generalizable knowledge and, as a result, must be reviewed and approved by a REC. This includes CRTs conducted outside health care settings, such as in education or public health research.

Health research must be reviewed in a manner that ensures that ethical issues receive appropriate consideration. Studies vary in the magnitude and complexity of ethical issues posed. As a result, RECs ought to undertake a proportional approach to the review of study protocols. According to this approach, CRTs that pose substantial risk or involve vulnerable participants ought to receive intensive scrutiny; in contrast, CRTs that pose low risk and do not involve vulnerable participants may be eligible for an expedited or delegated review.

### Identifying Research Participants

The clear identification of research participants is central to the implementation of protections outlined in national and international ethics guidelines. Research participants are those most directly affected by the conduct of research, and researchers and RECs have an obligation to protect the interests of research participants. We offer four criteria to guide the appropriate identification of human research participants in a CRT based on a defining feature of research participants [18]. The criteria attempt to precisely delineate whose interests are sufficiently directly affected that they ought to be considered research participants.

**Recommendation 3: Researchers should clearly identify the research participants in CRTs. A research participant can be identified as an individual whose interests may be affected as a result of study interventions or data collection procedures, that is, an individual (1) who is the intended recipient of an experimental (or control) intervention; or (2) who is the direct target of an experimental (or control) manipulation of his/her environment; or (3) with whom an investigator interacts for the purpose of collecting data about that individual; or (4) about whom an investigator obtains identifiable private information for the purpose of collecting data about that individual. Unless one or more of these criteria is met, an individual is not a research participant.**

The first criterion refers to individuals who are the intended recipients of a study intervention. This includes health professionals targeted by an educational intervention designed to promote evidence-based practice and patients targeted by a new therapy for a disease.

The second criterion refers to individuals who are directly targeted by an intervention delivered at the cluster level. This includes patients in a CRT investigating alterations of health delivery systems. It does not, however, include patients in a CRT of an educational intervention delivered to health professionals with the aim of promoting evidence-based practice. As argued in more detail elsewhere [18], simply being a patient of a professional participating in a CRT of an educational, knowledge translation, or quality improvement intervention does not make one a research participant. Although the professional may have received an intervention aimed at improving practice, the professional is still expected to act in the best interests of his or her patients and in accordance with professional practice standards—the loyalty of the health professional to the patient remains intact. Therefore, the welfare interests of the patients of a health care provider participating in a CRT are not jeopardized.

In some CRTs, clusters in the control arm are allocated to usual practice or no treatment, i.e., individuals may be neither recipients nor targets of any study interventions. However, when individuals in the experimental arm of the study are considered research participants, individuals in the control arm ought to be considered research participants, as their interests may be affected by lack of access to the study intervention or other appropriate care or benefit, and thus, they are entitled to protection (see recommendation 12).

The third and fourth criteria refer to individuals who provide data by interacting with investigators (e.g., through focus groups, interviews, or additional examinations) or individuals about whom investigators obtain identifiable private information (e.g., through review of patient health records). Individuals whose data are provided to the research team in anonymized form (e.g., from administrative data sources or registries) are not considered research participants.

Many CRTs involve cluster members who do not meet the criteria for research participants. For example, if the study intervention is designed to promote evidence-based practice by health professionals, and does not directly intervene in patient care, and if the researchers do not interact with patients or collect their identifiable private information, then those patients are not research participants [18].

### Obtaining Informed Consent

**Recommendation 4: Researchers must obtain informed consent from human research participants in a CRT, unless a waiver of consent is granted by a REC under specific circumstances.**

The obligation to obtain informed consent stems from the ethical principle of respect for persons, which requires that the choices of autonomous individuals be respected [19]. To be valid, such choices must be sufficiently informed, voluntary, and considered. Therefore, unless a waiver of consent is granted by a REC (see recommendation 6), researchers must seek the informed consent of potential research participants (or their proxy decision makers), and may enroll only those participants who provide consent.

In the informed consent process, researchers must provide potential participants with adequate information about the purpose of the study, study interventions and data collection procedures, the potential benefits and risks of study participation, and alternatives to participation. The aim is to enable participants to make a reasonable determination about whether enrolling in the study is consistent with their preferences and values. Detailed disclosure requirements are enumerated in international and national research ethics guidelines. Generally, informed consent refers to randomization, study interventions, and data collection procedures. However, in some CRTs different participants may need to provide consent to different elements. For example, health professionals as the recipients of an educational intervention may need to consent to study interventions, whereas patients may need to consent to data collection.

**Recommendation 5: When participants' informed consent is required, but recruitment of participants is not possible before randomization of clusters, researchers must seek participants' consent for trial enrollment as soon as possible after cluster randomization—that is, as soon as the potential participant has been identified, but before the participant has undergone any study interventions or data collection procedures.**

To be consistent with the moral purpose of informed consent, researchers should strive to identify participants and seek their consent before cluster allocation. In CRTs where identification and recruitment is not possible before randomization of clusters, participants may be legitimately enrolled following randomization of clusters. Researchers should, however, seek a potential participant's consent for study interventions and data collection procedures as soon as possible after the participant has been identified, and before administering any study interventions or data collection procedures. Seeking consent in this way after randomization is consistent with the moral purpose of informed consent, as potential participants may still freely choose whether or not to enroll in the trial [19].

Although seeking consent after randomization is consistent with the moral purpose of informed consent, researchers should be aware that selection biases can arise in such cases and should adopt design strategies that minimize the risk of bias [21],[28]–[30].

**Recommendation 6: A REC may approve a waiver or alteration of consent requirements when (1) the research is not feasible without a waiver or alteration of consent, and (2) the study interventions and data collection procedures pose no more than minimal risk.**

If seeking consent for study interventions or data collection procedures is not feasible (whether before or after randomization), researchers should apply for a waiver or alteration of consent, provided that study and data collection procedures pose no more than minimal risk to research participants. In a waiver of consent, the REC removes the requirement to obtain informed consent; in an alteration of consent, the REC permits the alteration or deletion of some of the standard elements of disclosure in the informed consent. Minimal risk refers to the risks of daily life, and includes the risks associated with routine physical examinations and review of medical records. Additional examples of study interventions and data collection procedures that pose only minimal risk are enumerated in the research ethics literature and ethics guidelines [31],[32].

As stated previously, many CRTs pose only low risk to research participants. The burden of demonstrating adequately to the REC that (1) obtaining informed consent is infeasible and (2) study participation poses only minimal risk falls to the researcher. Feasibility will depend on a variety of factors including cluster size, proximity of cluster members (and thus ease of contact), complexity of the consent process, research infrastructure (such as number of local health workers available to approach cluster members), and research funding.

Some researchers may be concerned that information provided to potential participants during the consent process will lead to bias that would undermine the interpretability of study results [33]. Rather than using a waiver of consent to address such concerns, the researcher and REC should consider an alteration of the consent process (such as blinding participants to their allocation status). Alternatively, researchers may consider adopting design features that address concerns about study validity while still adequately protecting participants' interests. For example, in an incomplete block design, each arm receives an intervention and simultaneously serves as a control for the other arm [34],[35]; participants in both study arms are provided with similar information during the consent process, thus equalizing any nonspecific (Hawthorne) effects across the study arms. Researchers should be aware that different consent procedures in the intervention and control arms of the trial may lead to bias [29],[30].

If obtaining informed consent is feasible for some but not all study interventions or data collection procedures, then researchers should obtain separate informed consent, where possible, for each procedure. For instance, in a CRT involving a cluster-level public health intervention for which a waiver of consent for the study intervention has been obtained, informed consent for data collection procedures may nonetheless be required.

In cases in which a waiver of consent has been granted, researchers and RECs may nonetheless consider making information about the study available to the eligible study population. This might occur, for example, via distribution of leaflets, poster placement in locations such as schools or physicians' offices, or public health bulletins. However, distribution of information about the study should not be construed as satisfying the requirement for informed consent. Rather, it is an additional step that researchers and RECs may pursue to demonstrate respect for persons when informed consent is not possible.

**Recommendation 7: Researchers must obtain informed consent from professionals or other service providers who are research participants unless conditions for a waiver or alteration of consent are met.**

Many CRTs deliver an intervention to professionals or other service providers (e.g., physicians, midwives, teachers) in order to produce an effect on cluster members (e.g., patients, students). These professionals or service providers are research participants and entitled to ethical protections. This includes the requirement for researchers to obtain their informed consent, unless a waiver of consent is granted by a REC (see recommendation 6).

It has been argued that health professionals have an obligation to participate in research that may improve patient care [36]. This prima facie moral obligation may indeed provide health professionals with a reason to agree to study participation when approached for informed consent. It does not, however, obviate the need to obtain their informed consent in specific CRTs when they are research participants. Consent discussions with these participants should include career-related risks, including risks due to detection of negligence or incompetence. Data about professional or provider performance should be kept confidential within the research team, unless circumstances arise that mandate disclosure to a professional certifying or licensing body.

Conditions for a waiver or alteration of consent may be met in a variety of circumstances involving professionals in CRTs (see recommendation 6). Provided the requirement of minimal risk is met, these circumstances include the following: when the number of professionals allocated to study interventions makes obtaining their informed consent infeasible in terms of either logistics or resources required; when cluster-level interventions mean that the professional cannot meaningfully refuse the study intervention (as when study interventions are delivered to entire health care teams as a unit); and when the researchers have grounds to believe that incomplete uptake of the study intervention or information provided to potential research participants during the informed consent process would threaten the validity of the trial results.

### Gatekeepers

Gatekeepers are individuals or bodies who may be called upon to protect the group-based interests that are affected by enrollment in a CRT [20]. Due to the challenges in obtaining individual informed consent in CRTs, researchers have historically turned to gatekeepers to perform a variety of roles, including providing proxy consent on behalf of individual cluster members, and giving permission to enroll clusters in trials.

**Recommendation 8: Gatekeepers should not provide proxy consent on behalf of individuals in their cluster.**

Legitimate proxy consent requires that the proxy decision maker be well acquainted with the potential research participant's values and beliefs, making the proxy decision maker well situated to make decisions consistent with the potential participant's wishes or interests. Further, proxy decision making is typically employed when the potential participant is incapable of making the decision for him- or herself. In CRTs, neither of these conditions is met, and so gatekeepers are not in a position to provide legitimate proxy consent on behalf of individual cluster members [20].

**Recommendation 9: When a CRT may substantially affect cluster or organizational interests, and a gatekeeper possesses the legitimate authority to make decisions on the cluster or organization's behalf, the researcher should obtain the gatekeeper's permission to enroll the cluster or organization in the trial. Such permission does not replace the need for the informed consent of research participants, when it is required.**

Gatekeepers may play an important role in the protection of cluster interests. When a CRT may have a substantial effect on a cluster or organization, obtaining the permission of a gatekeeper is one means of protecting the interests of the cluster or organization. For example, in a school-based trial, the school principal acting as a gatekeeper may provide permission to enroll a school in the CRT, after considering the impact on the school including availability of staff, financial implications of participation, and the likelihood that teachers or students would be willing to participate.

Gatekeepers may provide or withhold permission to enroll a cluster only when they have legitimate authority to do so. The legitimacy of gatekeepers' authority depends on the extent to which the following conditions are met: (1) their role within the cluster or organization endows them with the authority to make decisions on behalf of the cluster, e.g., they hold a political office or an administrative position within an organization that clearly gives them the relevant decision making authority, and (2) cluster members recognize the gatekeeper's authority. In situations where cluster members do not clearly accept the gatekeeper's authority to make the particular decision about enrollment, the legitimacy of that authority is questionable. Although a gatekeeper may legitimately give permission for cluster participation, gatekeeper permission is not a substitute for the informed consent of individual research participants in a CRT.

Some CRTs can have multiple levels of gatekeepers, e.g., one gatekeeper with authority over several clusters, but each cluster also having its own gatekeeper. Researchers and RECs should strive to identify situations in which the interests of different stakeholders within a CRT may conflict. For instance, the interests of an organization (such as a health care organization or school board) may conflict with the interests of clusters within that organization (such as physician practices or schools) or with the interests of individual cluster members (such as patients or students). Whereas requiring permission from a gatekeeper (such as an administrative head, board of governors, or school board) may serve to protect some stakeholders' interests, that gatekeeper may not be in a position to consider the interests of all stakeholders. Researchers and RECs should consider and, where possible, seek to safeguard the interests of all individuals or groups who may be affected by study interventions in a CRT.

The decision by a gatekeeper to withdraw a cluster from an ongoing CRT may have serious consequences for the participants as well as for the scientific validity of the study. Accordingly, researchers should do what they can to ensure that gatekeepers are unlikely to have reason to withdraw their cluster. Where possible, CRTs should be designed to minimize the effect of cluster withdrawal on study validity.

**Recommendation 10: When CRT interventions may substantially affect cluster interests, researchers should seek to protect cluster interests through cluster consultation to inform study design, conduct, and reporting. Where relevant, gatekeepers can often facilitate such a consultation.**

Gatekeepers may facilitate consultation between researchers and cluster members about the goals, design, and implementation of the study, as well as consultation about the research findings before they are disseminated [20]. Mechanisms for cluster consultation may include open public fora, community advisory boards, meetings with opinion leaders, presentations at religious or civic organizations, and the use of radio, television, or the Internet. These activities may help to protect and promote group interests by subjecting the study to examination and discussion by those whose interests may be affected or by some set of individuals who are familiar with those whose interests may be affected. Whether and to what extent cluster consultation needs to be undertaken will depend on the particular circumstances of the study. Recommendations from cluster consultation are not binding, and, where there are good reasons to do so, researchers may decline to make suggested changes to a study.

### Assessing Benefits and Harms

Establishing what constitutes a reasonable balance of harms and benefits is a central issue in research ethics. Component analysis provides researchers and RECs with a systematic approach to the ethical analysis of study benefits and harms (see Text S3 for general ethical principles) [37].

In CRTs evaluating different models of health service delivery, public health promotion campaigns, and educational interventions, “therapeutic procedures” often do not offer the participant the prospect of therapeutic benefit as it is traditionally understood in the medical sense. Instead, they offer different types of benefits, such as educational benefit. Identifying therapeutic procedures in CRTs therefore requires a broader definition in which therapeutic procedure refers to any intervention that is part of the experimental intervention, including particular components of a multifaceted, complex intervention. Further, the analysis of the benefits and harms of CRTs must take into account the fact that CRTs often involve effects on groups, health systems, and society as a whole.

Central to clinical equipoise is uncertainty about the comparative benefits and harms of the intervention in the experimental arm versus the control arm, according to a community of experts [17]. In individual patient randomized trials of clinical interventions, the relevant evidence relates to the balance of likely benefits and harms that might be incurred by individual research participants. CRTs may address questions that focus on the effectiveness of interventions solely for individual patients, in which case standard clinical equipoise considerations apply; however, they may also address public health questions, health systems questions, and knowledge translation or quality improvement questions. These latter types of questions are of interest to a variety of stakeholders; this suggests that the relevant evidence in CRTs may be broader, and justification for CRTs may need to take potential effects on a range of stakeholders into account.

Component analysis usefully directs the attention of researchers and RECs to the justification of the study intervention, control conditions, and data collection procedures when considering the benefits and harms of a CRT.

**Recommendation 11: The researcher must ensure that the study intervention is adequately justified. The benefits and harms of the study intervention must be consistent with competent practice in the field of study relevant to the CRT.**

The ethical concept of clinical equipoise requires uncertainty about the comparative benefits of the intervention in the experimental arm versus the control arm, according to a community of experts. This means that the benefits and harms of the study intervention must be consistent with competent practice in the field of study relevant to the CRT. In a CRT, study interventions may offer benefits to individual participants (in which case standard clinical equipoise considerations apply), or they may potentially benefit the clusters, organizations, or communities to which the research participants belong. The risks of study interventions may be borne by a stakeholder who may not necessarily derive benefit. So, it is difficult to compare directly the risks and potential benefits of study interventions. Rather, the REC should ensure that study interventions are consistent with competent practice in the particular field of study relevant to the CRT, such as medical practice, public health, health policy, or education. This requires the REC to appeal to evidence and the opinion of expert practitioners in the relevant field.

Random assignment of study interventions is justified if there is uncertainty in the relevant community of experts as to the preferred practice. The community of expert practitioners varies depending on the type of research question. For instance, public health clinicians are the relevant community of expert practitioners for public health questions, and policy makers or analysts are the relevant expert community for health policy questions. The scope of evidence relevant to the benefit–harm analysis may be broad, for example, when outcomes such as equity or costs are key issues for the research question. In the preparation of the study protocol, researchers should undertake a detailed review of the evidence on benefits and harms of the study intervention. Further, researchers may provide evidence regarding uncertainty among the relevant community of expert practitioners about the comparative benefits of the intervention in the experimental arm versus the control arm.

**Recommendation 12: Researchers must adequately justify the choice of the control condition. When the control arm is usual practice or no treatment, individuals in the control arm must not be deprived of effective care or programs to which they would have access, were there no trial.**

When the control arm is usual practice or no treatment, individuals in the control arm must not be deprived of effective care or programs to which they would have access if there were no study being conducted. Delayed provision of the study intervention to the individuals in the control arm does not justify depriving them of access to effective care or programs to which they would otherwise have access. As a minimum, clinical equipoise requires that the control arm should be given usual care within the study context.

Researchers and RECs may consider whether the control arm should receive some form of augmented care. In the context of a pragmatic CRT [38] of health policy or knowledge translation that aims to inform local policy, however, augmented care in the control arm may interfere with the scientific validity of the study by increasing the chances of a false negative result, or reducing the study's generalizability [33]. Thus, researchers and RECs need to give careful consideration to the advantages and disadvantages of this approach.

When reviewing the study protocol, the REC should consider whether and when the control clusters will receive the study intervention if the study intervention is shown to be effective.

**Recommendation 13: Researchers must ensure that data collection procedures are adequately justified. The risks associated with data collection procedures must (1) be minimized consistent with sound design and (2) stand in reasonable relation to the knowledge to be gained.**

Data collection procedures, including interviews, surveys, additional physical examinations that are not part of standard care, review of medical records, and the collection of economic information, are unlikely to benefit individuals or clusters directly. Rather, data collection procedures may benefit society in terms of new knowledge gained from the study. Researchers must, therefore, minimize the risks associated with data collection procedures consistent with sound design, and ensure that these risks stand in reasonable relation to the knowledge to be gained.

Use of electronic medical record or administrative data sources is to be recommended, as the burden and risk of data collection is much reduced, provided that (1) the raw data are fully de-identified prior to reaching the researcher and (2) reliable procedures for preventing re-identification are maintained throughout the research process.

### Protecting Vulnerable Participants

Vulnerable research participants fall into one or more of four broad categories: (1) children, (2) incapable adults (i.e., adults unable to provide informed consent), (3) people at undue risk of harm as a result of study participation, and (4) people in subordinate positions within social or organizational structures. CRTs may legitimately include vulnerable participants, provided that adequate protections for them are in place. Standard protections for vulnerable participants are discussed in Text S3 and are outlined in various national and international ethics guidelines.

Including vulnerable participants in CRTs poses the special challenge that their presence within clusters may be hidden, and thus, investigators may fail to employ the required standard protections. The presence of vulnerable participants may go unnoticed for two reasons: first, clusters may contain within them a small proportion of vulnerable individuals within apparently less vulnerable groups; and second, there may be individuals in a cluster who are not normally thought of as vulnerable, but who are vulnerable by virtue of their cluster membership. Examples of such individuals are health service staff, teachers, or other employees who may feel pressured to participate in a CRT involving their institution.

**Recommendation 14: Clusters may contain vulnerable participants. In these circumstances, researchers and RECs must consider whether additional protections are needed.**

Researchers and RECs should be mindful of the possibility that clusters may contain a mix of vulnerable and non-vulnerable participants. Where applicable, RECs should ensure that proposed consent procedures are appropriate for vulnerable participants within the cluster, and that study benefits and harms to such individuals are acceptable. For instance, a CRT studying programs for community treatment of mental illness may affect people living in group homes for the mentally ill. For this vulnerable subgroup, the REC will wish to ensure that consent procedures, including capacity assessment and the appropriate use of substitute decision makers, are appropriate. The REC may consult with an independent advocate or committee representing group home clients to ensure that people living in group homes are not unduly burdened by changes in access to community services.

In some cases, the study intervention may run the risk of exacerbating preexisting inequalities within clusters [39]. Where applicable, the REC should take this potential adverse outcome into account in the assessment of study benefits and harms.

The presence of vulnerable participants within a cluster does not preclude the use of a waiver of consent for all human research participants in the cluster.

**Recommendation 15: When individual informed consent is required and there are individuals who may be less able to choose participation freely because of their position in a cluster or organizational hierarchy, RECs should pay special attention to recruitment, privacy, and consent procedures for those participants.**

Some CRTs are conducted in the setting of clusters or organizations in which some members may be less able to express a free choice about trial participation because of their position within the hierarchy. When investigators are recruiting or obtaining consent from these individuals, they should conduct informed consent negotiations in such a way as to limit the potential for coercive influence from cluster or organizational leaders. For instance, consent negotiations should be conducted without the presence of cluster or organizational leaders, and cluster or organizational leaders should not be informed of the identities of those who agree to or decline study participation.

Vulnerability of this type does not preclude the appropriate use of a waiver of consent.

## Discussion

This consensus statement aims to provide researchers and RECs with guidance on the ethical design and conduct of CRTs in health research. General ethical principles are broadly understood to govern the practice of health research, but their application is complicated by design features of CRTs. As part of a five-year research project preceding the consensus process, the research team documented the reporting of ethical issues in CRTs and sought the perspectives of trialists and REC chairs on the ethical challenges they faced in the design, review, and conduct of CRTs. Background papers supplied detailed ethical analyses of the issues identified in this work, including the identification of research participants, informed consent, the role of gatekeepers, the balance of benefits and harms, and the protection of vulnerable participants. This combination of empirical work and ethical analysis provided the consensus process with a rich foundation, and helped ensure that the resulting consensus statement is reasonably comprehensive. Given the rapidly expanding nature of the field, however, we expect that significant revisions and additions to this document will be needed over the next five years.

The consensus statement is the result of a transparent and robust consensus process. The research team strived for transparency by publishing the project protocol and background papers in *Trials* (an open-access journal), posting project materials on a publically accessible wiki, providing a free webcast of the open portion of the consensus meeting, and holding an open consultation on the draft consensus statement with point-by-point response to all comments raised. The formal consensus process was led by a chair who is experienced in guideline development. Careful attention was paid to the small group processes of the expert panel to minimize the risk of psychosocial biases. Expert panel members were encouraged to question, comment on, and debate issues throughout the consensus process. Two drafts of the consensus statement were circulated to expert panel members after the closed meeting, which allowed members to raise any outstanding concerns they felt they had not been adequately addressed previously. Anecdotally, many expert panel members commented favorably on the quality of the consensus process.

The consensus statement is subject to limitations common to consensus processes. The research team identified participants from a variety of sources to represent the range of constituencies and perspectives that it felt should be represented. However, not all invitees agreed to participate, and some perspectives were underrepresented. For example, the expert panel was to include a trialist and a REC chair from a low- or middle-income country (LMIC), but a REC chair who could participate was not identified. As a result, LMIC perspectives were underrepresented. The authors believe that the consensus statement's applicability to CRTs in LMIC settings is supported by a number of factors, including consideration of LMIC examples in the background papers, the inclusion in the expert panel of six trialists with extensive experience in LMIC settings and members with expertise in the ethics of research in LMICs, and extensive discussion of LMIC issues in the closed meeting. As greater representation from LMICs could nonetheless have brought issues to the fore that were not considered, we recommend that subsequent revisions include greater LMIC representation.

## Supporting Information

Text S1 **Glossary of terms.**
(PDF)

Text S2 **Members of the Ottawa Ethics of Cluster Randomized Trials Consensus Group.**
(PDF)

Text S3 **General ethical principles.**
(PDF)

## Abbreviations

CRT: cluster randomized trial
LMIC: low- or middle-income country
REC: research ethics committee
