# Supplementary material for: The Ottawa Statement on the Ethical Design and Conduct of Cluster Randomized Trials
Source: PLoS Med. 2012 Nov 20;9(11):e1001346. doi: 10.1371/journal.pmed.1001346 (PMC3502500; doi:10.1371/journal.pmed.1001346)
Supplement: Text S1 — Glossary of terms. (PDF) [file pmed.1001346.s001.pdf]

## S1: Glossary of Terms

**Beneficence:** The ethical principle identifying the moral obligation not to harm needlessly, and when possible, to promote the welfare of research participants. In the context of clinical research, beneficence gives rise to the moral obligation to provide research participants with a reasonable balance of harms and benefits.

**Cluster Randomized Trial:** A study design that randomizes to different study arms groups or clusters of individuals (such as households, primary care practices, hospital wards, classrooms, neighbourhoods or communities), rather than independent individuals. Another distinguishing feature of CRTs is that the units of allocation, intervention, observation, and analysis may be different within a single study. CRTs may also be referred to as group randomized, place randomized, or community intervention trials.

**Clinical Equipoise:** The state of honest, professional disagreement among the community of experts about the preferred policy or practice for a particular problem. The research team identified the question: "Does clinical equipoise apply to CRTs?" as one of six ethical issues that needs to be addressed in CRTs. In individually randomized trials, questions about harms and benefits are addressed in part by the ethical requirement of clinical equipoise. Since CRTs may not involve physician-researchers and patient-participants, the applicability of clinical equipoise to CRTs is uncertain. In our background papers, we argue that clinical equipoise may be usefully grounded in a trust relationship between the state and research participants, and, as a result, clinical equipoise is applicable to CRTs.

**Cluster:** A group of individuals who share common interests or are associated institutionally, socially, geographically, or in time. Examples of clusters include households, medical practices, hospital wards, schools, neighbourhoods, and communities.

**Cluster Member**: Any individual who belongs to a cluster, regardless of status as a research participant or role in the CRT.

**Component Analysis:** A systematic approach to the ethical analysis of benefits and harms in research according to which therapeutic procedures and non-therapeutic procedures are evaluated separately. Therapeutic procedures must fulfill the requirement of clinical equipoise. The risks of non-therapeutic procedures must be minimised consistent with sound scientific design and stand in reasonable relation to the knowledge to be gained from the study.

**Control:** That to which a study intervention is being compared, including usual care or no intervention. Some CRTs may compare two interventions in a head-to-head comparison; in such cases there may be no control.

**Data Collection Procedures:** Means within the study used to collect information to answer the scientific question at hand. Examples of data collection procedures include interviews, surveys, additional physical examinations, or the collection of information from medical records or routine administrative sources.

**Evidence-based Practice**: The conscientious, explicit, and judicious use of current best evidence in making decisions about the care of individual patients, health or education policy, or service delivery.

**Explanatory Trial:** See under Pragmatic Trial (below).

**Gatekeeper:** Gatekeepers are individuals or bodies who may be called upon to protect the interests of clusters, organizations, or communities that are the setting for CRTs. Some CRTs can have multiple levels of gatekeepers, e.g., there might be one gatekeeper with authority over several clusters, but each cluster also having its own gatekeeper. Gatekeepers may protect group interests in a CRT by facilitating cluster consultation or by providing permission for the group to be enrolled in the study. However, permission from a gatekeeper to conduct a CRT that involves a particular group is not a substitute for individual informed consent.

The expert panel discussed use of the term “gatekeeper”, and such variants as “guardian” and “cluster representation mechanism”. However, the Panel concluded that the term “guardian”, which implies a formal status relationship, as exists between a parent and child or guardian and incapable adult, does not apply to cluster heads. The term “cluster representation mechanism” is problematic because “representation” may imply a relationship that confers greater and broader decisional authority than is appropriate.

**Incomplete Block Design:** An incomplete block design can be used to equalise non-specific effects (such as Hawthorne effects) between study arms and minimise their impact on the estimated intervention effect. In the simplest incomplete block design, the 2x2 balanced incomplete block design, each arm receives an intervention and serves as a control for the other arm. For example, in a guideline implementation trial, family practices randomized to one arm might receive an intervention for the management of asthma, while family practices randomized to the other arm might receive an intervention for the management of angina. Both arms contribute data about the intervention as well as the control condition. As both arms experience the same level of intervention, the Hawthorne effect should be equalized between the study arms.

**Interests:** The goods that an individual or group would ordinarily seek to protect, including health, welfare, economic, legal, and privacy.

**Justice:** The ethical obligation to distribute the benefits and burdens of research fairly. Justice gives rise to the need to protect vulnerable participants in research, and to compensate research participants who are harmed as a result of research enrollment.

**Legitimate Authority:** Refers to the power vested in an individual or body whose role within the cluster or organization endows them with the capacity to make decisions on behalf of the group. Only gatekeepers with legitimate authority may provide permission to enrol a cluster in a CRT. In situations where cluster members do not recognise the gatekeeper’s authority, the legitimacy of that authority is questionable.

**Minimal Risk:** Minimal risk refers to the risks of daily life, and includes the risks associated with routine physical examinations or psychological testing. Examples of study interventions and

data collection procedures that pose only minimal risk are enumerated in the research literature and ethics guidelines.

**Moral Status:** An individual or group with moral status is recognised as having interests that need to be taken into consideration and that determine whether or not they require protections. The moral status of communities, for example, is a matter of debate, in that opinions differ about whether and to what degree the interests of communities require ethical protections.

**Non-therapeutic Procedures:** Non-therapeutic procedures are interventions carried out purely for research purposes. They are performed in order to collect data, which will contribute to the evaluation of the outcome of a study. Non-therapeutic procedures include the review of medical records for data collection, additional clinical examinations solely for data collection purposes, diagnostic investigations such as blood tests or radiological investigations that have no bearing on clinical care but are solely to generate data, surveys, interviews, and focus groups.

**Pragmatic Trial**: A pragmatic trial seeks to determine whether the treatment works in “real world” conditions, by selecting typical participants, settings, and comparison treatments. In contrast, an **Explanatory Trial** seeks to determine whether the treatment works under “ideal” conditions, by imposing tight restrictions on participants, treatments, and settings.

**Private Information:** Personal information that has been collected with reasonable expectation of privacy. Personal information includes any factual or subjective information, recorded or not, about an identifiable individual. This includes information in any form, such as age, name, ID number, income, ethnic origin, blood type, opinions, evaluations, comments, social status, disciplinary actions, employee files, credit records, or medical records.

**Research Participant:** For the purposes of determining ethical protections, any individual whose interests may be affected as a result of study interventions or data collection procedures, that is, an individual (1) who is the recipient of an experimental (or control) intervention; or (2) who is the direct target of an experimental (or control) manipulation of his/her environment; or (3) with whom an investigator interacts for the purpose of collecting data about that individual; or (4) about whom an investigator obtains identifiable private information for the purpose of collecting data about that individual.

Note that our use of the term 'participant' is not intended to imply active participation; in some CRTs, e.g., when there is a cluster level intervention and a waiver of consent is approved by a REC, research participants may have little or no active role to play in the study.

**Respect for Persons:** The ethical principle requiring that researchers take seriously the choices of autonomous people, that is, people who can responsibly make their own decisions, and protect those who are incapable of making their own choices. This principle is the source of the moral rules of informed consent and confidentiality.

**Respect for Communities:** The ethical principle that investigators have an obligation to respect communal values, protect and empower communities, and, where applicable, abide by the decisions of legitimate communal authorities.

**Study Intervention:** A medical treatment, policy change, educational intervention, or complex intervention that is being evaluated in a CRT.

**Therapeutic procedures:** Therapeutic procedures are generally recognised as interventions administered with therapeutic warrant. More specifically, therapeutic procedures include any intervention that is being evaluated in the trial that offers the prospect of direct benefit to research participants. They often involve the same treatment or diagnostic interventions that a physician administers to her patient in the course of standard care. In addition, interventions in the control arm of a trial which are undertaken for the purposes of comparison with the active arm should be considered therapeutic procedures. These can include interventions that are ordinarily part of standard practice, placebos, or sham interventions, so long as they are included in the control arm for the purposes of comparison.

**Vulnerability:** The condition of diminished ability to protect one's own interests in decisions about research participation, which may allow for exploitation by others. Vulnerable populations may include children, incapable adults (i.e., adults unable to provide informed consent), people at undue risk of harm as a result of study participation (e.g., pregnant women), or people in subordinate positions within social or organizational structures (e.g. prisoners, military personnel). CRTs may also include (1) vulnerable individuals within apparently less vulnerable groups, or (2) individuals who are not normally thought of as vulnerable but become vulnerable because of their cluster membership.
