# Supplementary material for: The Ottawa Statement on the Ethical Design and Conduct of Cluster Randomized Trials
Source: PLoS Med. 2012 Nov 20;9(11):e1001346. doi: 10.1371/journal.pmed.1001346 (PMC3502500; doi:10.1371/journal.pmed.1001346)
Supplement: Text S2 — Members of the Ottawa Ethics of Cluster Randomized Trials Consensus Group. (PDF) [file pmed.1001346.s002.pdf]

S2: Members of the Ottawa Ethics of Cluster Randomized Trials Consensus Group

Expert Panel

Fernando Althabe MD, MSc
Trialist
Director, Department of Mother and Child Health Research, Institute for Clinical Effectiveness and Health Policy; Professor of Public Health, School of Public Health, University of Buenos Aires Medical School, Buenos Aires, Argentina

Allan Donner PhD, FRSC
Biostatistician, Research Team
Professor of Epidemiology and Biostatistics, Western University, London, Ontario, Canada

Geneviève Dubois-Flynn PhD
Research Funder
Manager and Senior Advisor of the Canadian Institutes for Health Research Ethics Office, Ottawa, Canada

Martin P. Eccles MD, FMedSci, FRCP, FRCGP, FFPHM
Trialist, Journal Editor, Research Team, Writing Group, Panel Chair
Professor of Primary Care Research and of Clinical Effectiveness, Newcastle University, Newcastle upon Tyne, UK

Sarah Edwards PhD
Bioethicist
Senior Lecturer in Research Ethics and Governance; Centre for Philosophy, Justice and Health, University College London, UK

Diana Elbourne PhD
Trialist
Professor of Healthcare Evaluation, London School of Hygiene and Tropical Medicine, London, UK

Sandra Eldridge PhD
Biostatistician, Trialist
Professor of Biostatistics, Barts and The London School of Medicine and Dentistry, Queen Mary University of London, London, UK

David Forster JD, MA, CIP
Research Ethics Perspective
Chief Compliance Officer, Office of Compliance, Western IRB, Olympia, Washington, USA

Jeremy M. Grimshaw MBChB, PhD, FRCGP

Trialist, Journal Editor, Co-principal Investigator, Writing Group
Senior Scientist, Ottawa Hospital Research Institute; Professor of Medicine, University of Ottawa, Ottawa, Ontario, Canada

Melody Lin PhD
Regulator
Deputy Director, Office for Human Research Protections and Director, International Activities, Office for Human Research Protections, Department of Health & Human Services, Rockville, Maryland, USA

Elizabeth Loder MD, MPH
Journal Editor
Clinical Epidemiology Editor, BMJ and Associate Professor of Neurology, Harvard Medical School, Boston, Massachusetts, USA

Eileen S. Naughton JD
Policy Maker
Rhode Island Representative, National Institutes of Health Council of Public Representatives, Providence, Rhode Island, USA

Rex J. Polson LLM, MD, FRCP
Research Ethics Chair
Chair of West Midlands – Solihull Research Ethics Committee, and Consultant Physician, Solihull Hospital, Heart of England NHS Foundation Trust, UK

Raphael Saginur MD
Research Ethics Chair, Research Team
Chair, Ottawa Hospital Research Ethics Board; Chief of Infectious Diseases, Ottawa

Abha Saxena MD
Scientist
Executive Sectretary, Research Ethics Review Committee, World Health Organization, Geneva, Switzerland

Julie Spence MD
Research Ethics Chair
Past Chair, Research Ethics Board, Department of Emergency Medicine, St. Michael's Hospital, Assistant Professor, University of Toronto, Toronto, Ontario, Canada

Charles Weijer MD, PhD
Ethicist, Co-principal Investigator, Writing Group
Professor, Rotman Institute of Philosophy, Western University, London, Ontario, Canada

Gerald White BComm
Policy Perspective

Former Assistant Deputy Minister of Health in Newfoundland and Labrador, Member of Health Council of Canada, Canada

Merrick Zwarenstein MD, PhD
Trialist, Research Team
Senior Scientist, Institute for Clinical Evaluative Studies, Toronto, Ontario, Canada

Expert Discussants

Catarina Kiefe PhD, MD
Trialist, Journal Editor
Professor and Chair, Dept of Quantitative Health Sciences, University of Massachusetts Medical School, Worcester, Massachusetts USA

Jonathan Kimmelman PhD
Ethicist
Associate Professor, Biomedical Ethics Unit, McGill University, Montreal, Quebec, Canada

Kathleen Lohr MA, MPhil, PhD
Distinguished Fellow (Health Services Research)
RTI International, Research Triangle Park, North Carolina, USA

Research Team (not on the Expert Panel)

Ariella Binik PhD (c)
Research Team
Rotman Institute of Philosophy, Western University, London, Ontario, Canada

Judith Belle Brown MSW, PhD
Research Team
Professor, Department of Family Medicine, Western University, London, Ontario, Canada

Robert Boruch PhD
Research Team
Professor of Education and Statistics, Co-Director, Center for Research and Evaluation in Social Policy, University of Pennsylvania, Philadelphia, Pennsylvania, USA

Jamie C. Brehaut PhD
Research Team, Writing Group
Scientist, Ottawa Hospital Research Institute; Associate Professor, University of Ottawa, Ottawa, Ontario, Canada

Shazia Chaudhry MSc
Research Team
PhD candidate, Department of Epidemiology and Community Medicine, University of Ottawa, Ottawa, Canada

Antonio Gallo BSc
Research Team
Rotman Institute of Philosophy and Schulich School of Medicine and Dentistry, Western University, London, Ontario, Canada

Andrew McRae MD, PhD, FRCPC
Research Team, Writing Group
Research Director, Division of Emergency Medicine, University of Calgary, Calgary, Alberta, Canada

Monica Taljaard PhD
Biostatistician, Co-principal Investigator, Writing Group
Scientist, Ottawa Hospital Research Institute, Ottawa, Ontario, Canada

Angela White PhD
Research Team, Writing Group
Rotman Institute of Philosophy Postdoctoral Fellow in Bioethics, Western University, London, Ontario, Canada
