# Supplementary material for: The Ottawa Statement on the Ethical Design and Conduct of Cluster Randomized Trials
Source: PLoS Med. 2012 Nov 20;9(11):e1001346. doi: 10.1371/journal.pmed.1001346 (PMC3502500; doi:10.1371/journal.pmed.1001346)
Supplement: Text S3 — General ethical principles. (PDF) [file pmed.1001346.s003.pdf]

S3: General Ethical Principles

In this supporting document we present ethical principles that govern the design and conduct of health research generally. The recommendations in the Ottawa Statement consider the application of these ethical principles to CRTs.

All research involving human participants should be conducted in accordance with four fundamental ethical principles: respect for persons; beneficence; justice; and respect for communities. These principles are grounded in moral theories, the research ethics literature, and convention and are foundational for national and international ethics guidelines [1,2,3].

The principle of respect for persons requires that: (1) choices of autonomous people, that is, people who can responsibly make their own decisions, are given serious consideration; and (2) people lacking autonomy, such as young children or adults with advanced dementia, are entitled to protection. The principle of respect for persons is the source of the moral rules of informed consent and confidentiality. The researcher is generally obligated to obtain agreement from a research participant (or his or her proxy decision maker) for study enrollment. For informed consent to be valid, the research participant (or proxy) must have the cognitive capacity to make the choice, be so situated as to choose freely, have adequate information, and understand what is at stake in the decision. Informed consent may not be required when it cannot feasibly be obtained and study participation poses only minimal risk. Researchers must also take necessary steps to protect the confidentiality of the research participant's personal information.

The principle of beneficence obliges researchers not to inflict unnecessary harm and, where possible, to promote the good of research participants. Health research often contains a mixture of study procedures, some offering reasonable prospect of benefit to research participants (therapeutic procedures), whereas others are administered solely to allow the scientific question to be answered (non-therapeutic procedures). Examples of therapeutic procedures include drug treatments, clinical examinations, or diagnostic interventions administered to patients in the course of standard medical care. Examples of non-therapeutic procedures include the review of medical records for data collection, additional clinical examinations solely for data collection purposes, and surveys.

According to a systematic approach to the ethical analysis of benefits and harms in research called component analysis [4], therapeutic and non-therapeutic procedures must be considered separately. Therapeutic procedures are justified if they satisfy clinical equipoise, which requires that they meet the standard of care for medical practice. In other words, there must be a state of honest, professional disagreement in the community of expert practitioners as to the preferred treatment. Non-therapeutic procedures, which generally offer no direct benefit to the research participant, are acceptable if the risks associated with them are minimised consistent with sound scientific design, and reasonable in relation to the knowledge to be gained. When the study involves a vulnerable population, such as children or incapable adults, the risks posed by non-therapeutic procedures must not exceed a minor increase above minimal risk. According to component analysis, one may conclude that the benefits and harms of a study are acceptable only when the moral rules for both therapeutic and non-therapeutic procedures are satisfied.

Because the distinction between therapeutic and non-therapeutic procedures may not always be clear in CRTs (see introduction to the section on "Assessing harms and Benefits"), we refer to the “study intervention”, “control condition”, and “data collection procedures”.

The principle of justice may be defined as the ethical obligation to distribute the benefits and burdens of research fairly. Researchers have an obligation to ensure that the means used to select research participants are equitable. Researchers must neither exploit the vulnerable, nor exclude without good reason those who stand to benefit from study participation. In order for proposed eligibility criteria to be evaluated, each criterion must be accompanied by a clear justification in the study protocol. The inclusion of a vulnerable group (such as children, incapable adults, or prisoners) requires a clear justification to demonstrate they are not being targeted merely as a matter of convenience. Further, insofar as is possible and practicable, the study population ought to mirror the target clinical population. The historical exclusion of children, women, and racial minorities from the benefits of research has led to a variety of contemporary initiatives to promote their inclusion in clinical research. The principle of justice also requires that provisions be in place to compensate research participants who are harmed as a result of research enrollment [5].

The principle of respect for communities means that researchers have an obligation to respect communal values, protect and empower communities, and, where applicable, abide by the decisions of legitimate communal authorities. Generally, the researcher-community relationship ought to be viewed as a partnership. Depending upon the degree to which the research affects the community as a whole and the specific features of the community, researchers may productively involve community partners throughout the research process, from defining the study question through the dissemination of results. Community consent to research participation ought to be restricted to cases in which the community leader is a legitimate authority who is empowered to speak on behalf of community members. Community consent does not replace the need for the informed consent of individual research participants [6].

References

[1] National Commission for the Protection of Human Subjects of Biomedical and Behavioral Research (1978) The Belmont Report: Ethical Principles for the Protection of Human Subjects of Biomedical and Behavioral Research. Washington: U.S. Government Printing Office.
[2] World Medical Association (2008) Declaration of Helsinki.
[3] Council for International Organizations of Medical Sciences (CIOMS) (2002) International Ethical Guidelines for Biomedical Research Involving Human Subjects. Geneva: CIOMS.
[4] Weijer C, Miller PB (2004) When are research risks reasonable in relation to anticipated benefits? Nature Medicine 10(6):570–573.
[5] Childress JF (1976) Compensating injured research subjects: the moral argument. Hastings Center Report 6(6): 21–27.
[6] Weijer C, Emanuel EJ (2000) Protecting communities in biomedical research. Science 289(5482):1142–1144.
